# The evaluation of clinical outcomes assessments and digital health technologies in clinical trials for obesity

**DOI:** 10.1186/s41687-025-00841-0

**Published:** 2025-02-20

**Authors:** Iris A. Goetz, Carolyn Sutter, Traci Abraham, Chisom Kanu, Kristina S. Boye, Tara Symonds

**Affiliations:** 1 Eli Lilly and Company, Lilly Corporate Center Indianapolis, Indianapolis, IN 46285 USA; 2 Clinical Outcomes Solutions, 53 West Jackson Boulevard, Chicago, IL 60604 USA; 3 Clinical Outcomes Solutions Ltd, Unit 68 Basepoint, Shearway Business Park, Shearway Road, Folkestone, UK

**Keywords:** Chronic weight management, Clinical trials, Obesity, Clinical outcome assessments, Patient reported outcomes, Performance outcome, Digital health technologies, Health-related quality of life, Physical function

## Abstract

**Background:**

Clinical trials for obesity have traditionally focused on weight loss and resolution of comorbidities as primary outcomes. However, secondary outcomes, such as the impact of weight reduction on patient experience, like health-related quality of life (HRQoL), have increasingly been recognized as important. Therefore, a review was conducted to determine the Clinical Outcome Assessments (COAs) and Digital Health Technologies (DHTs) used in clinical trials for obesity to assess the patient experience.

**Methods:**

Two clinical trial databases (United States & European Union) were reviewed to identify Phase 2–4 clinical trials for obesity (2018–2023). A targeted literature review was also conducted using the OVID database to identify clinical trial for obesity publications which included COAs/DHTs (2010–2023).

**Result:**

Trials from the databases (*n* = 53) and publications (*n* = 42) were included in data extraction (*N* = 73). This resulted in identification of 108 COAs, the majority being patient-reported outcome (PRO) measures (*n* = 83), but also 24 performance outcomes (PerfO) measures, and 1 composite PRO-clinician-reported outcomes (ClinRO) measure, as well as 2 DHTs. The most frequently identified PRO measures were the Short Form 36 and the Impact of Weight on Quality of Life Lite Clinical Trials. Twenty-four PerfO measures were also identified, with the 6-minute walk test being most common. These measures were most often used to construct secondary endpoints, with physical function (PF) being the most frequently specified domain. PRO measures assessing eating-related thoughts/behaviours, physical activity, and disordered eating were also frequently included, although individual measures varied widely across trials.

**Conclusion:**

Review of COAs and DHTs in registered clinical trials and publications for obesity found that PRO measures were the most common type of COA used to develop endpoints with current use of DHTs limited. Specifically, the physical function domain of multidimensional patient-reported outcome measures assessing health-related quality of life were often used to construct secondary endpoints. Further work is warranted to assess how the COAs and DHT data collected in clinical trials are viewed by regulators and payers.

**Supplementary Information:**

The online version contains supplementary material available at 10.1186/s41687-025-00841-0.

## Background

Obesity continues to represent a significant health challenge for populations across the globe. Since the 1980s, the prevalence of overweight and obesity has doubled globally. Currently, about one-third of individuals in the world could be categorized as overweight or obese [1].

Scientific evidence demonstrates strong associations between obesity and both morbidity and mortality, specifically with increased levels of particular cancers, hypertension, stroke, diabetes mellitus, and disability [2, 3]. As such, clinical trials for obesity have traditionally been concerned with weight loss and resolution of comorbidities as primary outcomes [4]. However, over the last 10 years, drug development programs have increasingly incorporated outcomes reported as most important by patients, including the effect of weight loss on health-related quality of life (HRQoL) and well-being. Additionally, patient satisfaction with treatment has become a point of importance [5]. Consequently, over the last several years, clinical outcome assessments (COAs) and other patient focused measures have been integrated into obesity trials to better measure patient satisfaction and experience, such as in trials for the drugs liraglutide and semaglutide [6, 7].

A COA is defined by the United States Food and Drug Administration (FDA) as any measure that describes or reflects how a patient feels, functions, or survives [8]. There are 4 general types of COA measures: Patient-reported outcomes (PROs), Observer-reported outcomes (ObsROs), Clinician-reported outcomes (ClinROs), and Performance outcomes (PerfOs) [8]. A PRO measure is defined as any report on the condition of a patient’s health that is obtained directly from the patient and does not involve clinician or outside interpretation of the patient’s response [8]. PRO measures attempt to capture the treatment experience from the patient’s point of view. An ObsRO is an evaluation of observable signs and behaviors pertaining to a patient’s health condition by individuals who are commonly around the patient (family, caregivers, etc.) [8]. ClinRO measures are reports that are obtained directly from a trained clinician and convey their interpretation of events, signs, and behaviors related to the patient’s condition. A PerfO is an assessment obtained by asking a patient to complete an established standardized task, such as reading an eye chart [8].

Lastly, digital health technologies (DHTs) can also be used to administer COAs and are generally defined as systems that use computing platforms, connectivity, software and/or sensors to capture patient-focused data [9]. DHTs can collect a wealth of information, including about how a patient is functioning, and therefore could be considered another type of COA, although not formally established by regulatory authorities as such. One example of how a DHT can be used to construct an endpoint is using a wearable fitness trackers to track steps taken per day as a measure of physical fitness. According to one recent scoping review, DHTs were most commonly used to collect physiological data (37.1%), clinical symptoms data (36.9%), and behavioral data (33.5%) [10].

Despite the increased use of COAs in clinical trials, there is no published overview in the literature describing their use to capture the patient perspective in clinical trials for obesity. This becomes particularly important as the patient’s perspective continues to be under assessed [11]. As such, the aim of this targeted literature review was to identify COAs and DHTs used in Phase 2–4 clinical trials for obesity during the 2018–2023 period to provide an overview of how the patient experience is being evaluated and their implementation to construct endpoints.

## Methods

Searches were performed using a 2-step process. The number of years and publications included in data extraction were limited to approximately *N* = 50 trials to stay within the scope of a targeted literature review.

Step 1 entailed a search of the 2 main United States (US) and European Union (EU) clinical trial databases (clinicaltrials.gov and clinicaltrialsregister.eu) to identify any COAs or DHTs used in obesity trials from June 2018 to June 2023. Searches were conducted using the combined key words: chronic weight management OR obesity OR weight loss OR overweight. Additionally, the parameters of adult only, drug treatment (for US searches only), and Phase 2–4 clinical trials were specified. Trials identified from the search were then screened for inclusion if they mentioned use of COAs or DHTs, with US trials screened first and only unique trials from the EU search included (with duplicates with the US results excluded).

Step 2 consisted of a targeted review of published literature between 2010 and 2023 which described clinical trials for obesity and included COAs/DHTs. The search of published literature was performed using the OVID (EMBASE, Medline, and PsycINFO) database, and search terms were developed based on initial searches to facilitate the identification of the most relevant articles. Screening criteria for results of the OVID search was a 2-part process; first, a broad screen based on item titles and/or abstracts was applied, and then all shortlisted titles or abstracts from this broad screen were reviewed for final eligibility.

Similarly, a search was conducted of oral and poster abstracts from the conference proceedings of relevant organizations, including Obesity Week, the American Diabetes Association, the European Association for the Study of Diabetes, the International Society for Pharmacoeconomic and Outcomes Research, and the International Society for Quality of Life Research. This search allowed capture of relevant material which may have been presented by poster or oral presentation but was not yet published in the literature. To stay within the scope of the limited review (approximately *N* = 50 clinical trials), the abstracts search was limited to the past 3 years (January 2021 - June 2023).

Upon completion of the respective reviews, data was extracted and synthesized to effectively present information related to COAs and DHTs currently being used in clinical trials for obesity.

## Results

After screening, a total of 53 unique registered clinical trial entries (*n* = 48 clinicaltrials.gov, *n* = 5 clinicaltrialsregister.eu) were taken forward for data extraction. Trials included were in Phase 2 (6; 11%), Phase 2–3 (8; 15%), Phase 3 (26; 49%), and Phase 4 (13; 25%). From the 53 trials, 108 different COAs were identified, including 83 PRO measures (86%), 24 PerfO measures (22%), and 1 composite PRO-ClinRO measure (1%). Additionally, 2 DHTs were identified that were used to capture data for a performance outcome related to physical activity. Most trials specified at least 1 COA endpoint (*n* = 50 trials; 94%). Some trials designated results of COAs as more than 1 type of endpoint (e.g., both a primary and secondary endpoint for different phases of the same trial). The clinical trials investigated 33 drug treatments for obesity (see supplementary information for a full list of drug treatments included in clinical trials).

There were 33 publications identified from the literature search and 9 additional conference abstracts; a total of 42 publications were included for data extraction. After screening for duplicate trials, an additional 20 trials and 13 PRO measures were identified. Most sources were published from 2016 to 2023, with the majority (*n* = 28, 67%) published from 2021 onward. The published literature presented clinical trials exploring a variety of drug treatments similar to those seen in the database review.

Across the data extracted from the clinical trial entries and published literature, a total of 108 unique COAs, as well as 2 DHTs, were identified as being used to measure outcomes in obesity trials. There was a total of 73 clinical trials (*N* = 73). The majority of the COAs were PRO measures (*n* = 83; 77%), although many PerfOs (*n* = 24; 25%) were also identified, as well as 1 composite PRO-ClinRO measure (*n* = 1; 1%). The COAs were used to construct co-primary or key secondary endpoints in 25 trials (*n* = 34%), secondary or supportive secondary endpoints in 63 trials (86%), and exploratory endpoints in 8 trials (11%). The measures were organized into the following categories: HRQoL, mental health-related, disordered eating-related, eating-related thoughts and behaviors, physical activity, sleep-related, cognition-related, symptoms and impacts related to osteoarthritis, impacts on work, and “other” measures.

### Health related quality of life measures

Measures of HRQoL were most frequently used to derive endpoints in the 73 trials (*n* = 45/73; 62%); see Table 1. All of the identified measures assessing HRQoL (*n* = 7) were PRO measures.

The SF-36 (Short-Form 36 items) was the most frequently identified measure and was used to derive an endpoint for 32 of the 45 trials. The SF-36 is comprised of 8 domains (mental health, role emotional, social functioning, vitality, role-physical, PF, bodily pain, and general health) which contribute to 2 summary component scores of wellbeing (physical health and mental health) [12]. The SF-36 was mainly used to construct a secondary endpoint considering change from baseline to end of trial. Less often, it was used to form a confirmatory or supportive secondary endpoint considering percentage of patients who achieved a predefined meaningful within-person improvement, or to construct an exploratory endpoint. The endpoint position is specified in Table 1, below.

The IWQoL-Lite-CT was the second most widely used HRQoL measure and was used to construct a secondary endpoint for 21 trials. The IWQoL-Lite-CT is an alternative version of the IWQoL-Lite developed specifically for use in obesity clinical trials to assess psychosocial and physical functioning of patients. The measure has 20 items and 2 main domains (physical and psychosocial) [13, 14]. Seven of the 21 trials used only the IWQoL-Lite-CT physical function composite score as a confirmatory or secondary endpoint; the trials used either change in the composite score from baseline to end of trial or used the percentage of participants that experienced meaningful improvement for these endpoints.

Measures less frequently reported were the EQ-5D-5 L [15], which was used to form a secondary endpoint measuring mobility, self-care, usual activities, pain/discomfort and anxiety/depression. Additionally, the Mean QOL questionnaire and World Health Organization Quality of Life - Brief Version [16] were used to construct secondary endpoints, with the World Health Organization Quality of Life- Brief Version measuring physical health, psychological health, social relationships, and environment. Finally, the Treatment-Related Impact Measure-Weight [17] was used to derive a supportive secondary endpoint measuring HRQoL related to weight loss. Additional details about these measures are provided in Table 1, below.

**Table 1:** COAs identified in review: health-related quality of life

Measure	Type of COA	# of Trials (*n* = 45)	Concept	Endpoint Position	Frequency per Measure Across Trials
SF-36^a^	PRO	32	HRQoL (physical function, role-physical, bodily pain, general health, vitality, social functioning, role-emotional, mental health)	Secondary	20
				Confirmatory secondary	7
				Supportive secondary	2
				Key secondary efficacy	1
				Exploratory	2
IWQOL-Lite-CT	PRO	21	HRQoL (physical function, self-esteem, sexual life, public distress, work)	Secondary	16
				Confirmatory secondary	5
EQ-5D-5 L	PRO	9	QoL (mobility, self-care, usual activities, pain/discomfort and anxiety/depression)	Secondary	5
				Not provided	4
IWQOL-Lite	PRO	7	HRQoL (physical function, self-esteem, sexual life, public distress, work)	Secondary	2
				Supportive secondary	1
				Exploratory	4
TRIM-Weight	PRO	1	QoL related to weight loss	Supportive secondary	1
Mean QOL questionnaire	PRO	1	QoL	Secondary	1
WHOQOL-BREF	PRO	1	QoL (physical health, psychological health, social relationships, environment)	Secondary	1

EQ-5D-5 L EQ-5D 5 Level; COA = clinical outcome assessment; QoL = quality of life; IWQoL-Lite = Impact of Weight on Quality of Life-Lite; IWQoL-Lite-CT = Impact of Weight on Quality of Life-Lite Clinical Trials; PRO = patient-reported outcome; SF-36 = Short-Form 36 items; TRIM-Weight = Treatment-Related Impact Measure-Weight; WHOQOL-BREF = World Health Organization Quality of Life-BREF

^a^As it is possible that sponsors did not distinguish between use of the SF-36 and SF-36 v2, we have reported all use of these measures as “SF-36” in the table

*Note*: Sponsors are not required to include all secondary and exploratory trial endpoints when listing information on US or EU websites, thus measures included in a trial without endpoint information are listed as “not provided” as it is possible the measure was included as a secondary or exploratory endpoint although not reported on the website

### Mental health-related measures

A total of 8 mental health related measures were identified across 21 trials (*n* = 21/73; 29%) (See Table 2). Most of the mental health-related measures(*n* = 7/8; 88%) were PRO measures; additionally, 1 composite PRO-ClinRO measure (the Columbia Suicide Severity Rating Scale) was identified.

The Patient Health Questionnaire 9 items (PHQ-9) [18] was used in 14 of the 21 trials as a measure of depression, most frequently to derive a secondary endpoint, with some trials specifically indicating its use to construct a supportive secondary endpoint, whilst others used it to construct a safety outcome. The PHQ-9 is a PRO measure that aims to assess severity of depression in patients. This questionnaire contains 9 items corresponding to the 9 criteria on which the DSM-IV-based diagnosis of depression and depression-related disorders. These items include interest/pleasure in doing things, feeling depressed or hopeless, sleep difficulty, energy level, appetite, self-image, concentration ability, moving or speaking slowly, and suicidal thoughts [19].

Less frequently used measures of depression for deriving secondary endpoints were the Columbia Suicide Severity Scale, Beck Depression Inventory second edition, Patient Reported Outcomes Measurement Information System Depression and Anxiety scales, the Generalized Anxiety Disorder 7, and the Self-reporting Questionnaire 20-item. The Perceived Stress Scale [20] was also identified but the endpoint positioning was not described. Additional details about these measures are described in Table 2, below.

**Table 2:** COAs identified in review: mental health-related

Measure	Type of COA	# of Trials (*n* = 21)	Concept	Endpoint Position	Frequency of Measure Across Trials
PHQ-9	PRO	14	Depression	Secondary	5
				Supportive secondary	4
				Other secondary	1
				Exploratory	1
				Safety outcome	2
				Not provided	1
C-SSRS	PRO & ClinRO	6	Depression	Secondary	4
				Safety outcome	3
BDI-II	PRO	4	Depression	Secondary	2
				Screening	1
				Not described	1
PSS	PRO	3	Stress	Exploratory predictor variable	1
				Not described	2
PROMIS Anxiety (Short Form v1.08a)	PRO	1	Anxiety	Secondary	1
PROMIS Depression (Short Form v1.08a)	PRO	1	Depression	Secondary	1
GAD-7	PRO	1	Anxiety	Exploratory predictor variable	1
SRQ-20	PRO	1	Distress	Secondary	

BDI-II = Beck Depression Inventory 2nd Edition; COA = clinical outcome assessment; C-SSRS = Columbia Suicide Severity Rating Scale; GAD-7 = Generalized Anxiety Disorder 7; PHQ-9 = Patient Health Questionnaire-9; ND = Not disclosed; PRO = patient-reported outcome; PROMIS = Patient Reported Outcomes Measurement Information System; PSS = Perceived Stress Scale; SRQ-20 = Self-Reporting Questionnaire 20-item

*Note*: Sponsors are not required to include all secondary and exploratory trial endpoints when listing information on US or EU websites, thus measures included in a trial without endpoint information is listed as “not provided” as it is possible the measure was included as a secondary or exploratory endpoint although not reported on the website

### Eating-related thoughts and behaviors measures

A total of 25 measures of eating-related thoughts and behaviors were used in 18 trials (*n* = 18/73; 25%) (see Table 3). Most of these measures (*n* = 19/25; 76%) assessing eating-related thoughts and behaviors were PRO measures, although some PerfO measures (*n* = 6/25; 24%) were also used.

**Table 3:** COAs identified in review: eating-related thoughts and behaviors

Measure	Type of COA	# of Trials (*n* = 18)	Concept	Endpoint Position	Frequency of Measure Across Trials
VAS	PRO	11	Appetite sensations/Palatability	Co-primary	2
				Secondary	10
				Exploratory	1
				Not described	1
CoEQ	PRO	4	Food cravings (craving control, craving for savory, craving for sweet, positive mood)	Secondary	1
				Supportive secondary	1
				Exploratory	2
PFS	PRO	3	Responsiveness to food environment	Secondary	1
				Exploratory predictor variable	1
				Not described	1
EI	PRO	3	Eating behavior (cognitive restraint, dietary disinhibition)	Supportive secondary	1
				Exploratory predictor variable	1
				Not described	1
LFPQ	PerfO	3	Food preference	Secondary	2
				Supportive secondary	1
FFCS	PRO	2	Food cravings	Secondary	2
Chocolate Milkshake Drinking Task	PRO	2	Hedonic food intake	Co-primary	2
FCQ-T	PRO	1	Food cravings	Supportive secondary	1
FCQ-T-Reduced	PRO	1	Food cravings	Exploratory predictor variable	1
PFS-15 item	PRO	1	Responsiveness to food environment	Secondary	1
FCI	PRO	1	Food cravings	Secondary	1
ASA24	PRO	1	Diet quality	Co-primary	1
DFS	PRO	1	Diet quality	Co-primary	1
3-Day Food Diary	PRO	1	Diet quality	Co-primary & Secondary	1
GLMS	PRO	1	Food intensity perception	Co-primary	1
LHS	PRO	1	Food preference	Co-primary	1
BHE	PRO	1	Barriers (lack of knowledge, self-control, time)	Exploratory predictor variable	1
RED-13	PRO	1	Food reinforcement	Co-primary	1
DEBQ	PRO	1	Eating behavior (emotional eating, external eating, restraint)	Exploratory predictor variable	1
TFEQ	PRO	1	Eating behavior	Not described	1
FSI	PerfO	1	Satiety	Secondary	1
Becker DeGroot Markov Auction Task, modified	PerfO	1	Food reinforcement	Co-primary	1
Reinforcing Efficacy of High- and Low-calorie Food	PerfO	1	Food reinforcement	Exploratory predictor variable	1
RRV-F	PerfO	1	Motivation to eat	Secondary	1
4-Meter Fast Paced Walk Test	PerfO	1	Motivation to eat	Secondary	1

ASA24 = Automated Self-Administered 24-Hour Recall; BHE = Barriers to Healthy Eating and Physical Activity; COA = clinical outcome assessment; CoEQ = Control of Eating Questionnaire; DEBQ = Dutch Eating Behavior Questionnaire; DFS = Dietary Fat & Sugar Intake Questionnaire; EI = Eating Inventory; FCI = Food Craving Inventory; FCQ-T = General Food Cravings Questionnaire – Trait; FFCS = Favorite Food Craving Scale; FSI = Food Satiety Index; GLMS = General Labeled Magnitude Scale; LFPQ = Leeds Food Preference Questionnaire; LHS = Labeled Hedonic Scale; PerfO = performance outcome; PFS = Power of Food Scale; PRO = patient-reported outcome; RED-13 = Reward-Related Eating Questionnaire; RRV-F = Relative Reinforcing Value of Food; TFEQ = Three Factor Eating Questionnaire; VAS = visual analogue scale

*Note*: Sponsors are not required to include all secondary and exploratory trial endpoints when listing information on US or EU websites, thus measures included in a trial without endpoint information is listed as “not provided” as it is possible the measure was included as a secondary or exploratory endpoint although not reported on the website

Visual analogue scales (VAS) [21] measuring appetite sensations (including hunger, fullness, satiety, prospective food consumption) and palatability were most frequently identified, having been reported for 11 of the 18 trials. Most of the trials (10/11) used a VAS as to construct a secondary endpoint; less frequently, it was also a co-primary and exploratory endpoint. In general, Visual Analogue Scales are being used in clinical trials to measure concepts like pain, thirst, and hunger. To achieve this goal, scores are created from self-reported responses that are indicated by a written mark placed along a 10 cm line, where the left end of the line represents the absence of the concept, and the right end represents the highest amount of severity (worst pain, worst hunger, etc.) [21, 22]. Less frequently identified PRO measures are provided in Table 3, below. These PRO measures were primarily used to form a secondary endpoint but were also used to form a co-primary endpoint, and a supportive secondary endpoint, or to derive an exploratory predictor variable.

PerfO measures included the Reinforcing Efficacy of High- and Low-calorie Food, Leeds Food Preference Questionnaire, Becker DeGroot Markov Auction Task, Reward-Related Eating Questionnaire 13 item, the Relative Reinforcing Value of Food, and the 4-Meter Fast Paced Walk Test. These measures were used to derive co-primary endpoints, secondary endpoints, supportive secondary endpoints, and/or as an exploratory predictor variable. Additional details about each of these measures is provided in Table 3, below.

### Physical activity-related measures

A total of 9 measures of physical activity were identified in 16 trials (*n* = 16/73; 22%) (see Table 4). These measures assessing physical activity included 5 PRO measures (*n* = 5/9; 56%), 2 PerfO measures (*n* = 2/9; 22%), and 2 DHTs (*n* = 2/9; 22%).

The 6-Minute Walk Test (6-MWT) [23], a PerfO measure whereby distance covered in 6-minutes is calculated, was most frequently used among the measures (8/16 trials), having been used in 4 trials to construct a secondary endpoint and in 1 of the 4 trials also to form part of a co-primary endpoint with the Kansas City Cardiomyopathy Questionnaire (KCCQ). When used to form a co-primary endpoint, the 6-MWT was listed third in a hierarchical composite that also included all-cause mortality and heart failure events. The reported unit was the total “wins” for each treatment group obtained from a hierarchical comparison of the components (randomization to study completion). The endpoint position was not described for 4 trials.

The Patient Global Impression of Severity for physical function and the Patient Global Impression of Change for physical function were both equal in frequency of use to the 6-MWT, however, no endpoint positions were provided for either of these measures. The Patient Global Impression of Severity is a single item self-administered measure that aims to evaluate severity of condition from the patient’s perspective, and is commonly used as an outcome measure for various diseases [24]. The Patient Global Impression of Change is another self-administered measure that seeks to assess the perception of change following treatment (improvement versus worsening) from the point of view of the patient [25].

Less frequently identified measures in the trials database and published literature included the Paffenbarger Physical Activity Questionnaire, International Physical Activity Questionnaire, and the Exercise Self Efficacy Scale. These PRO measures were used to derive a primary endpoint, secondary endpoint, exploratory endpoint, and/or as an exploratory predictor variable. The 4-Meter Fast Paced Walk Test, a PerfO measure, was also identified and used to derive a secondary endpoint.

The 2 DHTs included the ActiGraph wGT3X-BTLink accelerometer, which was used to measure physical activity, gait, and balance, and the VitalCare digital health platform application, which was used to measure steps, calories per day, and exercise sessions per week, were used to construct part of a co-primary endpoint or a secondary endpoint. Additional details about each of these measures is described in Table 4, below.

**Table 4:** COAs and DHTs identified in clinical: physical activity-related

Measure	Type of COA	# of Trials (*n* = 16)	Concept	Endpoint Position	Frequency of Measure Across Trials
6MWT / 6MWD	PerfO	8	Physical capacity	Primary, Secondary	1
				Co-primary, Secondary	1
				Secondary	1
				Confirmatory secondary, Supportive secondary	1
				Confirmatory secondary	1
				Not provided	4
PGIS for physical activity/ function	PRO	8	Physical activity/function	Not provided	8
PGIC for physical activity/ function	PRO	8	Physical activity/function	Not provided	8
PPAQ	PRO	1	Physical activity	Exploratory	1
iPAQ	PRO	1	Physical activity	Screening	1
ESES	PRO	1	Exercise self-efficacy	Exploratory predictor variable	1
4-Meter Fast Paced Walk Test	PerfO	1	Physical capacity	Secondary	1
ActiGraph wGT3X-BTLink accelerometer	DHT	1	Physical activity, gait, balance	Co-primary	1
VitalCare digital health platform (app)	DHT	1	Physical activity (steps, calories per day, exercise sessions per week)	Secondary	1

6MWT = 6-minute Walk Test; 6MWD = 6-minute Walk Distance; COA = clinical outcome assessment; DHT = digital health technology; ESES = Exercise Self Efficacy Scale; iPAQ = International Physical Activity Questionnaire; PerfO = performance outcome; PGIC = Patient Global Impression of Change; PGIS = Patient Global Impression of Severity; PPAQ = Paffenbarger Physical Activity Questionnaire; PRO = patient-reported outcome

*Note*: Sponsors are not required to include all secondary and exploratory trial endpoints when listing information on US or EU websites, thus measures included in a trial without endpoint information is listed as “not provided” as it is possible the measure was included as a secondary or exploratory endpoint although not reported on the website

### Disordered eating-related measures

A total of 10 other measures of disordered eating (food addiction, impulsivity regarding food, and binge eating) were found for 15 trials (*n* = 15/73; 21%) (See Table 5). All of these measures assessing disordered eating (*n* = 10) were PRO measures.

The Eating Disorder Examination Questionnaire (EDE-Q 6.0) [26] was most frequently reported as a measure of disordered eating (11/15 trials) and was typically used to construct a secondary endpoint across the clinical trials (8 out of 11 trials), and in 1 trial to derive a supportive secondary endpoint. The endpoint position for this PRO measure was not provided for 1 trial. The EDE-Q 6.0 is a widely used self-reported measure that assesses behavior and attitudes in eating disorders [27]. This measure is based on the well-established Eating Disorder Examination (EDE), which has commonly been considered the gold standard for measuring eating disorders (ED) [28]. As it is self-reported and relatively simple to administer, the EDE-Q 6.0 offers a valid cost-efficient alternative to the EDE that can be particularly useful when dealing with large populations [29]. The EDE-Q was used to assess change in global score and change in all of the subscales (dietary restraint, eating concert, weight concern, and shape concern).

Less frequently identified PRO measures included the interview version of the EDE; the Yale Food Addiction Scale; the Urgency, Premeditation, Perseverance, Sensation Seeking, and Positive Urgency Impulsive Behavior Scale; the Binge Eating Scale; the Barratt Impulsiveness Scale; the Behavioral Inhibition/Activation Scale; the Eating Disorder Inventory; the Questionnaire on Eating and Weight Patterns; and the Eating Loss of Control Scale. These PRO measures were primarily used to construct secondary endpoints, but also derived co-primary endpoints, supportive secondary endpoints, or were used to form an exploratory predictor variable. Additional details about each of these measures is described in Table 5, below.

**Table 5:** COAs identified in review: disordered eating-related

Measure	Type of COA	# of Trials (*n* = 15)	Concept	Endpoint Position	Frequency of Measure Across Trials
EDE-Q 6.0	PRO	11	Disordered eating	Secondary	8
				Supportive secondary	1
				Not provided	2
EDE-I	PRO	8	Disordered eating	Co-primary	7
				Secondary	6
				Not provided	1
YFAS	PRO	6	Food addiction	Secondary	3
				Exploratory	1
				Exploratory predictor variable	1
				Not provided	1
UPPS-P	PRO	2	Impulsive behavior (urgency, deliberation, persistence, sensation seeking)	Secondary	2
BES	PRO	2	Binge eating, including key behavioral (e.g., rapid eating, eating large amounts of food) and affective/ cognitive symptoms (e.g., guilt, feeling out of control or unable to stop eating) that precede or follow a binge	Safety outcome	1
				Not provided	1
BIS-11	PRO	1	Impulsive behavior (attention, motor, self-control, cognitive complexity, perseverance, cognitive instability, as well as attentional, motor, non-planning impulsiveness)	Exploratory predictor variable	1
BIS/BAS	PRO	1	Behavioral inhibition (reward responsiveness, drive, fun seeking)	Exploratory predictor variable	1
EDI	PRO	1	Disordered eating (drive for thinness, bulimia, body dissatisfaction, ineffectiveness, perfectionism, interpersonal distrust, interoceptive awareness, maturity fears)	Supportive secondary	1
QEWP-5	PRO	1	Binge eating	Exploratory predictor variable	1
LOCES	PRO	1	Behavioral, cognitive/dissociative, and positive/euphoric aspects of loss-of-control eating	Supportive secondary	1

Binge Eating Scale (BES); BIS-11 = Barratt Impulsiveness Scale; BIS/BAS = Behavioral Inhibition/Activation Scale; COA = clinical outcome assessment; EDE-I = Eating Disorder Examination Interview; EDE-Q 6.0 = Eating Disorder Examination Questionnaire; EDI = Eating Disorder Inventory; LOCES = Loss of Control Eating Scale; PRO = patient-reported outcome; QEWP-5 = The Questionnaire on Eating and Weight Patterns; UPPS-P = Urgency, Premeditation, Perseverance, Sensation Seeking, and Positive Urgency Impulsive Behavior Scale; YFAS = Yale Food Addiction Scale

*Note*: Sponsors are not required to include all secondary and exploratory trial endpoints when listing information on US or EU websites, thus measures included in a trial without endpoint information is listed as “not provided” as it is possible the measure was included as a secondary or exploratory endpoint although not reported on the website

### Measures of impacts on work

A total of 4 measures of impacts on work were identified in 6 trials (*n* = 6/73; 8%) (see Table 6). All 4 of the measures assessing impacts on work were PRO measures.

The Work Productivity and Activity Impairment Questionnaire-Specific Health Problem v2.0 [30] was most frequently reported (5/6 trials) and was used to construct a secondary endpoint. This questionnaire is a version of the Work Productivity and Activity Impairment questionnaire that aims to measure the impact of disease on work productivity and activity in the context of a specific health problem [30, 31]. The self-administered instrument consists of 6 questions that evaluate employment status, missed work time due to condition, total amount of time worked, and feelings about the condition’s effect on productivity and ability in work and outside of work over the last seven days [31].

Less frequently described PROs included the Stanford Presenteeism Scale and the Work Limitations Questionnaire 8-item and 25-item. The Work Limitations Questionnaire 8-item and 25-item were used to derive secondary endpoints. Additional details about each of these measures is described in Table 6, below.

**Table 6:** COAs identified in review: measures of impacts on work

Measure	Type of COA	# of Trials (*n* = 6)	Concept	Endpoint Position	Frequency of Measure Across Trials
WPAI: SHP	PRO	5	Impact of weight on work productivity (absenteeism, presenteeism, work productivity loss, activity impairment)	Secondary	4
				Not provided	1
SP-6	PRO	4	Health status and employee productivity	Not provided	4
WLQ-25	PRO	1	Impact on work (time management, physical demands, mental-interpersonal demands, output)	Secondary	1
WLQ-8	PRO	1	Impact of weight on work productivity (time management, physical tasks, mental or interpersonal tasks, and output tasks along with an index of overall at-work productivity loss)	Secondary	1

COA = clinical outcome assessment; PRO = patient-reported outcome; SP-6 = Stanford Presenteeism Scale; WLQ-8 = Work Limitations Questionnaire- 8 item version; WLQ-25 = Work Limitations Questionnaire- 25 item version; WPAI: SHP = Work Productivity and Activity Impairment Questionnaire Specific Health Problem V2.0

*Note*: Sponsors are not required to include all secondary and exploratory trial endpoints when listing information on US or EU websites, thus measures included in a trial without endpoint information is listed as “not provided” as it is possible the measure was included as a secondary or exploratory endpoint although not reported on the website

### Sleep-related measures

A total of 5 sleep-related measures were identified in 5 trials (*n* = 5/73; 7%) (Table 7). All 5 of the measures used in these trials were PRO measures.

The Epworth Sleepiness Scale (ESS) [32] and Functional Outcomes of Sleep Questionnaire (FOSQ) [33] PRO measures were most frequently reported, having both been identified in 2 of the 5 trials. The ESS is a self-administered measure that evaluates sleep disorders by asking patients to rate sleepiness from 0 to 3 (0 indicating a low chance of falling asleep) in 8 common situations [32, 34]. Each question is then scored and summed up to create a total score which indicates higher rates of sleepiness during the day [32]. The FOSQ also measures the impact of sleepiness on daily life, and is considered the gold standard among similar measures [35]. This self-administered measure has 30 items that evaluate the impact of excessive daytime sleepiness on patients’ physical, mental, and social functioning [33]. One trial included the ESS, FOSQ, and FOSQ-10 as secondary endpoints. The ESS was used to measure the percent of participants with ESS ≤ 10 as part of a composite, and the FOSQ to measure change in a hierarchical composite score including the FOSQ-10 item subset score as well as the FOSQ vigilance and activity level domain scores. The other trial used the ESS and FOSQ to construct a key secondary efficacy endpoints. Additional sleep-related measures included the FOSQ-10, the Satisfaction, Alertness, Timing, Efficiency, and Duration scale, measuring sleep health, the Bergen Insomnia Scale, and the Baseline sleep hours survey (unspecified). These PRO measures were used to derive a secondary endpoint, and/or to form an exploratory predictor variable. Additional details about each of these measures is described in Table 7, below.

**Table 7:** COAs identified in review: sleep-related

Measure	Type of COA	# of Trials (*n* = 5)	Concept		Endpoint Position	Frequency of Measure Across Trials
FOSQ	PRO	2	Outcomes of sleep	Key secondary		1
				Secondary		1
ESS	PRO	2	Sleepiness	Key secondary		1
				Secondary		1
RU-SATED scale	PRO	1	Sleep health (regularity, satisfaction, alertness, timing, efficiency, duration)	Secondary		1
FOSQ-10	PRO	1	Outcomes of sleep (activity level, vigilance, intimacy, sexual relationships)		Secondary	1
Bergen Insomnia Scale	PRO	1	Sleep		Not provided	1
Baseline sleep hours survey (unspecified)	PRO	1	Sleep		Exploratory predictor variable	1

COA = clinical outcome assessment; DHT = digital health technology; ESS = Epworth Sleepiness Scale; FOSQ = Functional Outcomes of Sleep Questionnaire; FOSQ-10 = Functional Outcomes of Sleep Questionnaire 10-item; PRO = patient-reported outcome; RU-SATED = Satisfaction, Alertness, Timing, Efficiency, and Duration scale

*Note*: Sponsors are not required to include all secondary and exploratory trial endpoints when listing information on US or EU websites, thus measures included in a trial without endpoint information are listed as “not provided” as it is possible the measure was included as a secondary or exploratory endpoint although not reported on the website

### Cognition-related measures

A total of 17 cognition-related measures were identified in 2 trials (*n* = 4/73; 5%) (see Table 8), all of which were PerfO measures These 17 PerfO measures were identified in only 1 trial each.

**Table 8:** COAs identified in clinical review: cognition-related

Measure	Type	# of Trials (*n* = 4)	Concept	Endpoint Position	Frequency of Measure Across Trials
Probabilistic-Feedback Reward Task (unspecified)	PerfO	1	Cognition	Co-primary	1
**Brief Neuropsychological Battery for Obesity (BNBO) Measures**					
Delay Discounting, Kirby	PerfO	1	Cognition (temporal discounting)	Co-Primary	1
Oral Reading Recognition Test	PerfO	1	Cognition (language decoding, reading)	Co-Primary	1
Penn Progressive Matrices Test	PerfO	1	Cognition (fluid intelligence)	Co-Primary	1
Penn Word Memory Test	PerfO	1	Cognition (verbal episodic memory)	Co-Primary	1
Relational Task	PerfO	1	Cognition (visual relational processing)	Co-Primary	1
Delay Discounting	PerfO	1	Cognition (reward sensitivity)	Secondary predictor variable (Phase 1), Secondary outcome (Phase 2)	1
Variable Short Penn Line Orientation Test	PerfO	1	Cognition (visuospatial processing)	Co-Primary	1
**Core Neuropsychological Measures for Obesity and Diabetes (NMOB) Measures**					
Digital Symptom Substitution	PerfO	1	Cognition (processing speed)	Co-Primary	1
Dimensional Change Card Sorting	PerfO	1	Cognition (cognitive flexibility, task-switching)	Co-Primary	1
Go/No-Go Task	PerfO	1	Cognition (response inhibition)	Co-Primary	1
Matrix Reasoning Task	PerfO	1	Cognition (general cognitive ability, non-verbal reasoning ability)	Co-Primary	1
Picture Sequence Memory	PerfO	1	Cognition (learning, memory)	Co-Primary	1
**Cambridge Neuropsychological Test Automated Battery (CANTAB) Measures**					
Delayed Matching to Sample Test	PerfO	1	Cognition (visuospatial memory)	Co-Primary	1
Intra-Extra Dimensional Set Shift Test	PerfO	1	Cognition (rule acquisition and reversal)	Co-Primary	1
Paired Associates Learning Task	PerfO	1	Cognition (episodic memory & new learning)	Co-Primary	1
Stockings of Cambridge Test	PerfO	1	Cognition (spatial planning)	Co-Primary	1

COA = clinical outcome assessment; PerfO = performance outcome; PRO = patient-reported outcome

*Note*: Sponsors are not required to include all secondary and exploratory trial endpoints when listing information on US or EU websites, thus measures included in a trial without endpoint information is listed as “not provided” as it is possible the measure was included as a secondary or exploratory endpoint although not reported on the website

The PerfO measures were used to construct co-primary endpoints, exploratory endpoints, and/or exploratory predictor variables. Additional details about each of these measures is described in Table 8, below.

### Measures of symptoms and impacts related to Osteoarthritis

A total of 10 measures of symptoms and impacts related to osteoarthritis were identified in 4 trials (*n* = 4/73; 5%) (see Table 9). All 10 of these were PRO measures.

The Western Ontario and MacMaster Universities Osteoarthritis Index [36] was most frequently reported (4/4 trials). This index is a self-administered instrument primarily used to measure physical function in patients with osteoarthritis of the knee and hip. The instrument has 24 questions which cover the areas of pain (5 questions), stiffness (2 questions), and physical function (17 questions). In 2 of the trials, this measure was used to derive a co-primary endpoint related to physical function and stiffness, while in the other trial it was used to construct a confirmatory secondary endpoint measuring pain, stiffness, and physical function.

Less frequently reported PRO measures included the Patient Reported Outcomes Measurement Information System - Pain, Arthritis Self-Efficacy Scale, Numeric Rating Scale, Knee Injury and Osteoarthritis Outcome Score, Intermittent and Constant Osteoarthritis Pain, the VAS knee pain, VAS physical function impacts due to knee pain, and VAS impact on daily life of knee pain, and the Pain Catastrophizing Scale. These measures were used to derive a co-primary endpoint and a secondary endpoint. Additional details about each of these measures is described in Table 9, below.

**Table 9:** COAs identified in review: symptoms and impacts related to osteoarthritis

Measure	Type of COA	# of Trials (*n* = 4)	Concept	Endpoint Position	Frequency of Measure Across Trials
WOMAC	PRO	4	Physical function, stiffness	Co-Primary	3
				Confirmatory secondary	1
ICOAP	PRO	1	Pain from osteoarthritis	Confirmatory secondary	1
KOOS	PRO	1	Symptoms of knee osteoarthritis	Co-Primary	1
ASES	PRO	1	Arthritis self-efficacy (pain, function, other symptoms)	Secondary	1
Pain NRS	PRO	1	Pain	Secondary	1
PCS	PRO	1	Pain catastrophizing (rumination, magnification, helplessness)	Secondary	1
PROMIS Pain (Short Form v1.08a)	PRO	1	Impact of pain on daily life	Co-Primary	1
VAS Pain	PRO	1	Pain due to knee pain	Not described	1
VAS Physical Function	PRO	1	Physical function due to knee pain	Not described	1
VAS Impact on Daily Life	PRO	1	Impact of knee pain on daily life	Not described	1

ASES = Arthritis Self-Efficacy Scale; COA = clinical outcome assessment; ICOAP = Intermittent and Constant Osteoarthritis Pain; KOOS = Knee Injury and Osteoarthritis Outcome Score, Intermittent and Constant Osteoarthritis Pain; NRS = Numeric Rating Scale; PRO = patient-reported outcome; PCS = Pain Catastrophizing Scale; PROMIS = Patient Reported Outcomes Measurement Information System; WOMAC = Western Ontario and MacMaster Universities Osteoarthritis Index

### Other measures

A total of 14 other measures covering a variety of conceptual domains were identified in 14 trials (*n* = 12/73; 16%) (see Table 10). Most of these (13/14) were PRO measures, with 1 PerfO measure (the Acetaminophen test) also identified.

Among these various types of measures, the KCCQ was frequently used (4/12 trials) to form both a secondary endpoint and a co-primary endpoint (based on the clinical summary score). The KCCQ is a disease specific instrument that primarily measures HRQoL in patients with congestive heart failure. The instrument contains 23 items that measure the areas of physical limitation, self-efficacy, social interference, quality of life, and symptoms [37].

The Weight Related Signs and Symptom Measure and International Consultation on Incontinence Questionnaire-Urinary Incontinence-Short Form were also used in 4 trials, although their endpoint positioning was not described. Less frequently reported PRO measures are summarized in Table 10 below. These measures were mostly used to construct secondary endpoints or exploratory predictor variables, in the case of the Weight Bias Internalization scale, Body Satisfaction Scale, Philadelphia Mindfulness Scale, Social Support Scale, Weight Efficacy Lifestyle Questionnaire and Weight Efficacy Lifestyle Questionnaire Short Form. In contrast, the Monell Forced Choice Test was used to derive a co-primary endpoint. Additionally, the 1 PerfO measure, the Acetaminophen test for gastric emptying, was used to derive a co-primary endpoint measuring gastric emptying. Additional details about each of the measures described above is provided Table 10, below.

**Table 10:** COAs identified in review: other measures

Measure	Type of COA	# of Trials (*n* = 12)	Concept	Endpoint Position	Frequency of Measure Across Trials
KCCQ	PRO	4	Symptoms and physical limitations associated with heart failure (symptom stability, frequency and burden, physical function, social limitation, self-efficacy, quality of life)	Co-primary, Secondary	4
WRSSM	PRO	4	Weight-related signs and symptoms	Not provided	4
ICIQ-UI-SF	PRO	4	Urinary incontinence	Not provided	4
WBIS	PRO	2	Weight bias internalization	Exploratory	1
				Not provided	1
Monell Forced Choice Test	PRO	1	Food preference (change in sweet/fat concentration)	Co-Primary	1
Body Satisfaction Scale	PRO	1	Body satisfaction	Exploratory	1
PHLMS	PRO	1	Mindfulness (present moment awareness, acceptance)	Exploratory predictor variable	1
GSRS	PRO	1	Gastrointestinal symptoms	Secondary	1
WHGQ	PRO	1	Hair growth	Not provided	1
Men’s Hair Growth Questionnaire	PRO	1	Hair growth	Not provided	1
Nail health survey (unspecified)	PRO	1	Nail growth	Not provided	1
Acetaminophen test	PerfO	1	Gastric emptying	Co-primary	1
Social Support Scale	PRO	1	Social support for healthy behavior	Exploratory predictor variable	1
WEL	PRO	1	Internal and external influences on self-efficacy related to weight	Exploratory predictor variable	1
WEL-SF	PRO	1	Internal and external influences on self-efficacy related to weight	Supportive Secondary	1

COA = clinical outcome assessment; KCCQ = Kansas City Cardiomyopathy Questionnaire; GSRS = Gastrointestinal Symptom Rating Scale questionnaire; ICIQ-UI-SF = International Consultation on Incontinence Questionnaire- Urinary Incontinence Short Form; PerfO = performance outcome; PHLMS = Philadelphia Mindfulness Scale; PRO = patient-reported outcome; WBIS = Weight Bias Internalization Scale; WEL = Weight Efficacy Lifestyle Questionnaire; WEL-SF = Weight Efficacy Lifestyle Questionnaire Short Form; WHGQ = Women’s Hair Growth Questionnaire; WRSSM = Weight-Related Sign and Symptom Measure

*Note*: Sponsors are not required to include all secondary and exploratory trial endpoints when listing information on US or EU websites, thus measures included in a trial without endpoint information is listed as “not provided” as it is possible the measure was included as a secondary or exploratory endpoint although not reported on the website

## Discussion

As previously mentioned, scientific evidence indicates strong associations between obesity and both morbidity and mortality, and individuals with obesity have a higher risk of certain illnesses (particular cancers, diabetes mellitus, disability, stroke, hypertension, as well others) than their non obese counterparts [2, 3]. Additionally, there is evidence that obesity is associated with lower HRQoL, and that even individuals with obesity who could currently be considered “healthy” may already be in transition to a future plagued by poor health [38]. The majority of therapies and treatments for obesity, regardless of approach, are concerned with addressing the abovementioned outcomes, however, determining the most appropriate endpoint for a treatment has remained a point of contention [39]. Often, because of its reliable and quantifiable nature, the reduction of body weight is used as a standard in therapies [40], and a focus is placed on the resolution of comorbidities. Nevertheless, many drug development programs for obesity are beginning to incorporate endpoints that are specifically geared toward measuring concepts considered most important by patients using COAs and DHTs. The purpose of this targeted literature review was to identify these measures in clinical trials for obesity as presented in clinical trial registrations from the past 5 years and related published literature and present a clear picture of which measures are being used to capture the patient experience, and how they are being implemented to construct endpoints within trials.

This targeted review identified a total of 108 COAs and 2 DHTs being used to measure outcomes in clinical trials for obesity. The majority of COAs were PRO measures (*n* = 83), although some PerfOs (*n* = 24) were also identified, as well as 1 composite PRO-ClinRO measure. Interestingly with the advent and increased use of DHTs, only 2 were reported in 2 trials.

A variety of concepts were measured using these COAs/DHTs, with measures of HRQoL most frequently included as secondary endpoints. Specifically, the SF-36 and IWQoL-Lite-CT were most consistently used to derive endpoints in the clinical trials. These measures were used to derive either confirmatory or supportive secondary endpoints, with the PF scores were most often used.

Measures of specific aspects of HRQoL, including mental health and physical activity were also included frequently in clinical trials for obesity, with measures of mental health often used as safety outcomes while physical activity measures were most often included to construct secondary endpoints. The PHQ-9 was consistently used as a measure of depression, while the 6-MWT was most frequently used as a measure of physical activity.

Outcomes related to eating-related thoughts and behaviors and disordered eating were also included across many trials. However, the measures used were generally not as consistent across these trials although similar concepts were measured such as disordered eating, binge eating, appetite or palatability, food cravings, food preferences or food reinforcement.

Less frequently, measures related to osteoarthritis and obstructive sleep apnea comorbidities were identified. Other outcomes related to work, cognition, social support, and comorbidities such as heart failure, incontinence, or hair growth were also included in some trials. Measures specific to weight loss treatment, the Treatment-Related Impact Measure-Weight, weight-related symptoms, or self-efficacy related to weight full and short form, were only identified in single trials or publications.

### Limitations

A limitation of this targeted review is that not all COA measures used to construct endpoints in the clinical trials were necessarily disclosed on the FDA/European Medicines Agency websites. Only primary and key secondary endpoints used in the endpoint hierarchy to power a study need to be disclosed. Often additional secondary endpoints and exploratory endpoints are not cited. This is borne out by the fact that more COAs were reported in the published literature than were disclosed during registration, suggesting an element of selection, most likely by the sponsor submitting the evidence and likely choosing the most directly impacted concepts in their endpoint hierarchy (i.e., PF) but still collected broader impacts for dissemination in publications. As reviewers at the EMA/FDA could only comment on what they are presented with by the sponsor, it is difficult to determine with certainty how other COAs may have contributed to registration success and market access. Due to the project’s limited scope, this targeted review was also limited to consideration of clinical trial registrations from the past 5 years and abstracts from the past 3 years. However, given the recent growth in obesity trials with inclusion of patient-centered outcomes it is likely most COAs and DHTs being consistently included in current trials were captured.

## Conclusion

Review of Clinical Outcome Assessment (COA) measures/Digital Health Technologies (DHTs) in registered clinical trials and publications for obesity found that Patient Reported Outcome (PRO) measures were the most common type of COA used to develop endpoints with current use of DHTs limited. Moreover, multi-dimensional PRO measures assessing HRQoL were most often used. Specifically, the SF-36 and IWQoL-Lite/IWQoL-Lite-CT generic and disease-specific (respectively) measures assessing health-related quality of life (HRQoL) have the most evidence of use in clinical trials for obesity. Most often, these measures, along with other HRQoL PRO measures, have been used in Phase 2–4 (most frequently in Phase 3) clinical trials and to construct secondary endpoints, usually considering outcomes associated with physical function. An interesting next step would be to investigate how COA data is viewed by regulators and payers to understand the importance of such data during regulatory interactions. Additional research is also needed to understand whether the most frequently used measures are considered adequate for assessing outcomes in clinical trials for obesity or whether new measures are required to more adequately assess the concepts of interest, especially with next generation treatments.

## Electronic supplementary material

Below is the link to the electronic supplementary material.

Supplementary Material 1

## Data Availability

Data and materials will be made available upon reasonable request.

## Abbreviations

ClinRO: Clinician-reported outcomes
COA: Clinical outcome assessment
DHTs: Digital health technologies
ED: Eating disorder
EDE: Eating Disorder Examination
EDE-Q: Eating Disorder Examination Questionnaire
EMA: European Medicines Agency
EOT: End of trial
ESS: Epworth Sleepiness Scale
EU: European Union
FDA: United States Food and Drug Administration
FOSQ: Functional Outcomes of Sleep Questionnaire
HRQoL: Health-related quality of life
IWQoL-Lite: Impact of Weight on Quality of Life-Lite
IWQoL-Lite-CT: Impact of Weight on Quality of Life-Lite Clinical Trials
KCCQ: Kansas City Cardiomyopathy Questionnaire
ObsRO: Observer-reported outcome
PHQ-9: Patient Health Questionnaire-9
PerfO: Performance outcome
PF: Physical function
PRO: Patient-reported outcome
SF-36: Short Form 36 Health Survey
US: United States
VAS: Visual Analogue Scale
WBIS: Weight Bias Internalization Scale
