# Supplementary material for: The evaluation of clinical outcomes assessments and digital health technologies in clinical trials for obesity
Source: J Patient Rep Outcomes. 2025 Feb 20;9:23. doi: 10.1186/s41687-025-00841-0 (PMC11842654; doi:10.1186/s41687-025-00841-0)
Supplement: Supplementary file 1 — Supplementary Material 1 [file 41687_2025_841_MOESM1_ESM.docx]

List of Drug Treatments Used in Trials

1. Bimagrumab
2. Bimagrumab + Semaglutide
3. Botulinum toxin type A
4. Bupropion
5. Cagrilintide
6. Cagrilintide + Semaglutide
7. Contrave
8. Fisetin
9. Hydroxychloroquine sulphate
10. Liraglutide
11. Lisdexamfetamine
12. Lisdexamfetamine Dimesylate
13. Lorcaserin
14. Lorcaserin, extended-release
15. Metformin
16. Metformin + Sibutramine
17. Muscle5 + TRIM7
18. Naltrexone + Bupropion (NB)
19. Orlistat
20. Phentermine
21. Phentermine + Topiramate
22. Phentermine + Topiramate extended-release
23. RiduZone
24. Saxenda
25. Semaglutide
26. Sibutramine
27. Tirzepatide
28. Tesofensine + Metroprolol
29. Topiramate, extended-release
30. TRIM7
31. Undisclosed
32. Undisclosed
33. Undisclosed
